# Modelled climatic suitability contraction and high-altitude persistence: Projected mid-century distribution dynamics of *Ophiocordyceps sinensis* across the Tibetan Plateau under a selected climate scenario

**DOI:** 10.1371/journal.pone.0354200

**Published:** 2026-07-21

**Authors:** Qiuyang Wei, Yuanchuan He, Shijiang Chen

**Affiliations:** 1 Chongqing University of Chinese Medicine, Chongqing, China; 2 Chongqing Academy of Chinese Materia Medica, Chongqing, China; Zhejiang Agriculture and Forestry University: Zhejiang A and F University, CHINA

## Abstract

*Ophiocordyceps sinensis*, a vulnerable fungal resource endemic to the Tibetan Plateau, faces climatically driven habitat degradation under ongoing climate change. Existing studies document its distribution but rarely address temporal dynamics under ongoing climate shifts. To fill this research gap, we implemented five-fold spatial block cross-validation (SBCV)-enhanced MaxEnt modelling and quantified extrapolation risks via Multivariate Environmental Similarity Surfaces (MESS; mean = 0.734 ± 0.135) using 201 spatially filtered occurrence points. Model performance was primarily evaluated via geographically independent spatial validation, yielding a mean test area under the receiver operating characteristic curve of 0.953 ± 0.002, confirming robust spatial transferability across the Tibetan Plateau. Integration of static topographic and dynamic bioclimatic variables across past–current–future timelines enabled validation against historical baselines (1970–2000), followed by Shared Socioeconomic Pathways 3–7.0 (SSP3–7.0) scenario projections. Elevation and warmest-quarter precipitation emerged as dominant predictors, collectively explaining 91.3% of habitat suitability. Under the BCC-CSM2-MR model and SSP3–7.0 scenario, conditional projections indicate severe near-term climatic suitability contraction (54% loss in modelled suitable habitat area relative to the 1970–2000 historical baseline, from 396,000 km^2^ to 182,000 km^2^ by 2021–2040), with over 82% of this total loss concentrated in marginal zones. Driven by localized expansion in the Tanggula Mountains that offsets low-elevation declines, the total modelled suitable habitat area stabilized at 198,000 km^2^, accounting for 3.78% of China’s land area, during the mid-century (2041–2060) under this model-scenario combination. This validated framework provides conditional insights for identifying potential climate refugia of *O. sinensis* and supporting evidence-based climatic suitability assessments.

## 1. Introduction

The Tibetan Plateau (TP), known as the Asian Water Tower and considered a regional amplifier of climate change impacts, sustains environmental security across East Asia through its ecological integrity [1]. Since the 1960s, accelerated warming at a rate of 0.35°C/decade-double the global average-has intensified ecosystem vulnerability [2]. These changes manifest as 15% glacial retreat since 1984, threatening Asia’s major river systems [3], while elevation-dependent warming forces endemic species like *Ophiocordyceps sinensis* to migrate upward at ~40 m/decade [4]. These cascading effects exacerbate extinction risks for climate-constrained species and threaten the stability of the plateau’s fragile biodiversity.

Endemic to the TP, *O. sinensis* is a rare medicinal fungus occupying a critical ecological and socioeconomic nexus [5]. Recognized as one of the world’s most valuable biological resources, it demonstrates significant pharmacological properties including immunomodulatory, antioxidant, and organ-protective effects (respiratory, renal, hepatic, neurological, and cardiovascular systems), with emerging evidence of antitumor, anticancer, and antiviral activities [6]. Consequently, its market value exceeds $20,000/kg, sustaining livelihoods for millions of Tibetan herders and forming the region’s second-largest economic sector after pastoralism [7].

However, *O. sinensis* is highly dependent on specific climatic and geographic conditions, with existing studies identifying core distribution constraints including altitude (3,500–4,500 m), warmest-season temperature (7.8–13.8°C), and wettest-season precipitation (200–600 mm) [8]. These thresholds collectively shape its spatial distribution. Under accelerating climate change, its climatically suitable habitat faces significant shrinkage, driving (1) upslope migration of suitable areas, and (2) degradation of marginal lowland suitable habitats [9]. Therefore, a systematic analysis of climate-driven distribution dynamics across historical, contemporary, and future periods is essential for the ecological conservation of this species, which is currently assessed as Vulnerable (VU) on the IUCN Red List of Threatened Species^TM^ [10].

In recent years, the Maximum Entropy model (MaxEnt) has been extensively employed to evaluate climate change impacts on species distributions, owing to its robust predictive accuracy and flexibility [11]. By integrating species occurrence records with environmental variables, MaxEnt quantifies habitat suitability across climate scenarios and identifies key distribution drivers [12]. For rare species such as *O. sinensis*, characterized by sparse occurrence data (often <50 records), MaxEnt still exhibits robust performance. Such robustness arises from its maximum entropy principle, which enables effective capture of environment-species correlations without reliance on large sample sizes [13]. While empirical studies indicate MaxEnt’s predictive accuracy stabilizes at approximately 50 training samples [14], the algorithm remains reliable for habitat-specialized, narrowly distributed taxa like *O. sinensis* even with smaller sample sizes. Support for this lies in studies on *Ophiocordyceps gracilis*, a congener of *O. sinensis* with analogous habitat traits, in which high predictive performance (Area Under the Curve, AUC > 0.95) was attained using only 24 occurrence points [15]. Such capability offers critical methodological support for modeling low-density species like *O. sinensis* across temporal scales, ranging from historical distribution reconstruction to future projection.

Although prior studies have examined both contemporary and future distributions of *O. sinensis*, a critical gap remains in understanding its multi-temporal distribution dynamics. We selected the Beijing Climate Center Climate System Model version 2 Medium Resolution (BCC-CSM2-MR) global climate model (GCM) and Shared Socioeconomic Pathway 3–7.0 (SSP3–7.0) scenario for future projections—justified by the model’s superior simulation of TP seasonal precipitation and elevation-dependent warming, the scenario’s alignment with observed TP climate trends [2], and comparability with prior *Ophiocordyceps* studies [7]. To address the gap, we developed a tiered framework focused on pivotal environmental drivers: (1) Reconstructing historical distributions using paleoclimate datasets by core parameter; (2) Quantifying current habitat fragmentation patterns; and (3) Simulating future range shifts under the selected SSP scenario.

## 2. Methods

### 2.1 Collection and screening of distribution points

**Fig 1:**
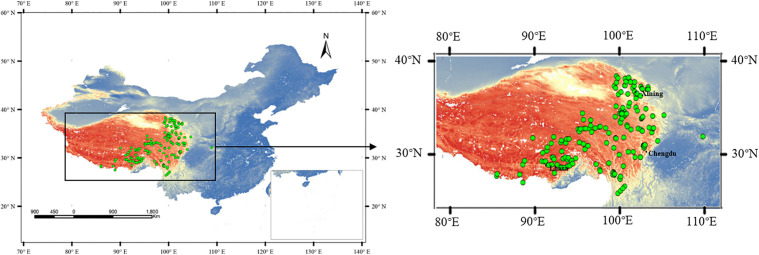
Spatially filtered occurrence records of *O. sinensis* in China. The map includes an inset overview of China, a core map of the Tibetan Plateau. All occurrence points shown are the final spatially filtered dataset used for model training and validation. Map from China’s Standard Map Service, Ministry of Natural Resources of the People’s Republic of China, with review approval number GS (2022) 1873.

To compile a comprehensive dataset of distribution records of *O. sinensis* across China, we integrated an extensive set of literature-sourced occurrences from ecological surveys [15,16] and referenced data from the Global Biodiversity Information Facility (GBIF, https://www.gbif.org), Discover Life (https://www.discoverlife.org), and the Species Diversity Data Platform (ESPECIES, http://www.especies.cn). After removing the distribution points outside of China and duplicate records, we applied spatial thinning with a 10 km minimum distance between points using ENMTools v.1.1.2 to reduce sampling bias [17], yielding 201 spatially independent occurrence points for modeling. These validated points were then imported into ArcMap 10.8 for downstream modeling. All maps utilize China’s Standard Map Service (GS (2022)1873) (Fig 1).

### 2.2 Environmental parameters

Climate, soil, and terrain were selected as the environmental factors for this study. Nineteen bioclimatic variables at 30-arcsecond resolution (~1 km^2^) were sourced from WorldClim (version 2.1, http://www.worldclim.org), representing key thermal and hydrological constraints on *O. sinensis* distribution. Historical climate data (observational baseline, 1970–2000) were obtained from the WorldClim v. 2.1 bioclimatic dataset. For near-term and future climate projections, we employed the BCC-CSM2-MR global climate model (developed by the Beijing Climate Center) participating in CMIP6 and featured in IPCC AR6. Under the SSP3–7.0 scenario, we extracted near-term (2021–2040) and mid-century (2041–2060) climate projection layers at a consistent 30-arcsecond resolution (~1 km^2^), ensuring spatiotemporal consistency across analyses.

Given *O. sinensis*’ edaphic lifecycle (infection of soil-dwelling host larvae, with development completed entirely within topsoil), soil variables were included to capture microhabitat constraints on host survival and fungal development [8]. While climatic factors dominate its large-scale distribution, edaphic variables define fine-scale habitat suitability, justifying their inclusion in the full model. The 16 pedological variables (0–30 cm depth) were sourced from the China Soil Map Based Harmonized World Soil Database (HWSD v2.0, https://data.tpdc.ac.cn/zh-hans/). The 3 topographic indices—elevation (alt), slope, and aspect—were extracted by ArcGIS with a spatial resolution of 30″. For future projections, soil and terrain parameters were held constant.

The initial 38 variables were spatially harmonized using a China boundary mask (GS (2022)1873) and standardized to 30-arcsecond resolution (Table S1 in S1 File). Variables were optimized using a three-step protocol: first, retaining ecologically relevant variables supported by previous studies [8,15]; second, excluding variables with <0.1% contribution from an initial MaxEnt run; third, removing multicollinear variables with |r| ≥ 0.8 (ENMTools v.1.1.2) while retaining the more ecologically meaningful and higher-contribution predictor in each pair. This process yielded 16 optimized predictors (bolded in Table 1), with the full correlation matrix provided in Table S2 and Figure S1 in S1 File.

**Table 1:** Environmental variables associated with *O. sinensis.*

Classification	index	description	units
Bioclimatic variables	**bio_1**	**annual mean temperature**	**°C × 10**
	bio_2	mean diurnal range	°C × 10
	**bio_3**	**isothermality**	**%**
	**bio_4**	**temperature seasonality**	**sd × 100**
	**bio_5**	**max temperature of warmest month**	**°C × 10**
	bio_6	min temperature of coldest month	°C × 10
	bio_7	temperature annual range	°C × 10
	bio_8	mean temperature of wettest quarter	°C × 10
	bio_9	mean temperature of driest quarter	°C × 10
	bio_10	mean temperature of warmest quarter	°C × 10
	bio_11	mean temperature of coldest quarter	°C × 10
	bio_12	annual precipitation	mm
	bio_13	precipitation of wettest month	mm
	bio_14	precipitation of driest month	mm
	bio_15	precipitation seasonality	coefficient of variation
	bio_16	precipitation of wettest quarter	mm
	**bio_17**	**precipitation of driest quarter**	**mm**
	**bio_18**	**precipitation of warmest quarter**	**mm**
	bio_19	precipitation of coldest quarter	mm
Soil data	t_gravel	topsoil gravel content	% weight
	t_sand	topsoil sand fraction	% weight
	**t_silt**	**topsoil silt fraction**	**% weight**
	t_clay	topsoil clay fraction	% weight
	t_ref_bulk	topsoil reference bulk density	kg/dm³
	**t_oc**	**topsoil organic carbon**	**% weight**
	**t_ph_h**_**2**_ **o**	**topsoil ph (h**_**2**_ **o)**	**-log (h +)**
	t_cec_clay	topsoil cec (clay)	cmol/kg
	**t_cec_soil**	**topsoil cec (soil)**	**cmol/kg**
	t_bs	topsoil base saturation	%
	t_teb	topsoil teb	cmol/kg
	**t_caco3**	**topsoil calcium carbonate**	**% weight**
	t_caso4	topsoil gypsum	% weight
	**t_esp**	**topsoil sodicity (esp)**	**%**
	**t_usda_tex**	**USDA soil texture classification**	**–**
	t_ece	topsoil salinity (elco)	dS/m
Terrain factors	**alt**	**elevation**	**m**
	**slope**	**slope**	**°**
	**aspect**	**aspect**	**°**

Note: Bolded variables represent the final optimized predictor set retained for MaxEnt modeling.

### 2.3 Model configuration and robustness assessment

Maximum Entropy modeling was implemented in MaxEnt v3.4.1, using 201 spatially filtered O. sinensis occurrence records and 16 optimized environmental predictors. To mitigate spatial autocorrelation, a 5-fold spatial block cross-validation (SBCV) framework (ENMTools v.1.1.2) was established as the primary approach for model tuning, robustness assessment and performance evaluation.

Non-overlapping 1° × 1° geographic grid blocks were generated to match the species’ spatial autocorrelation scale on the Tibetan Plateau, ensuring complete geographic independence between training and test folds. Specifically, the study area was divided into 24 non-overlapping 1° × 1° geographic blocks using ENMTools v.1.1.2. For each of the 5 cross-validation folds, blocks were assigned to each of the 5 folds, and for each fold, the blocks assigned to that fold served as the independent test set while the remaining blocks were used for training. This process was repeated across 5 folds with non-overlapping fold assignments. Background points (10,000) were randomly sampled from the study area (excluding occurrence localities) and stratified to ensure spatial coverage. Feature classes and regularization multiplier were tuned by selecting the combination that maximized mean test AUC across SBCV folds; the final model used linear, quadratic, and hinge features with a regularization multiplier of 1.0. Full parameters were provided in Table 2.

**Table 2:** Core parameter settings of 5-fold spatial block cross-validation.

Parameter	Specific Setting	Rationale for Setting
Spatial block generation tool	ENMTools v.1.1.2	Consistent with the spatial filtering tool used in occurrence data preprocessing, and in line with standard spatial validation protocols for species distribution models
Spatial block specification	1° × 1° non-overlapping geographic grid	Matches the spatial autocorrelation scale of *O. sinensis* distribution on the Tibetan Plateau, to ensure no geographic overlap and spatial autocorrelation interference between training and test sets
Number of cross-validation folds	5 folds	Balances sample size allocation and validation independence, with the test set of each fold covering heterogeneous geographic units of the species’ full distribution range
Sample allocation rule	For each fold, 1 geographic block is randomly selected as the independent test set, with all remaining blocks used as the training set, with 5 cycles covering all blocks	Ensures complete geographic independence of the training and test sets, and eliminates the sample non-independence bias of random splitting
Core basis for model tuning	Mean test AUC of 5-fold spatial block cross-validation	Ensures that the optimization of model hyperparameters is centered on spatial generalization ability
Uncertainty quantification	11 bootstrap replicate runs for each cross-validation fold	Quantifies the random error of model prediction, with final outputs averaged across all folds and replicates
Fixed model parameters	Maximum iterations: 500; Background points: 10,000; Output format: logistic	Consistent with the general specifications of the MaxEnt model, to ensure the reproducibility of analysis

Model tuning (regularization multipliers, feature classes) was based on the mean test AUC of 5-fold SBCV. Eleven bootstrap replicates were conducted per fold to quantify uncertainty, with outputs averaged across replicates and folds. Fixed parameters included 500 maximum iterations, 10,000 stratified random background points, and logistic output for habitat suitability [18].

Model robustness was assessed via four complementary approaches: (1) consistency of key performance metrics (AUC, omission rates, True Skill Statistics [TSS]) across SBCV folds and bootstrap replicates; (2) the relationship between mean prediction differences and block sample size across 24 geographic units to evaluate spatial stability; (3) Jackknife tests to quantify the relative contribution and non-redundant predictive information of each environmental variable [19].

### 2.4 Extrapolation risk and accuracy validation

To quantify the model’s extrapolation risk, MESS analysis was performed in Python 3.9 (Matplotlib 3.7, Scikit-learn 1.2) with a unified protocol, consistent with standard species distribution modeling practices [20]. For MESS similarity analysis, we adopted the 16 optimized environmental predictors retained in the final MaxEnt model, and integrated single-variable similarities through weighted summation—where weights were derived from each variable’s percent contribution to the MaxEnt model (Supplementary Material Table S3 in S1 File) to reflect their ecological relevance to *O. sinensis*. All environmental variables were first Z-score standardized based on training set statistics to eliminate unit bias and prevent data leakage; single-variable similarity was then calculated by comparing each point’s environmental conditions to the min–max range of the training dataset, and total MESS similarity was subsequently obtained via weighted summation of these single-variable values, with MESS scores ranging from -∞ (extreme environmental novelty) to 1 (complete environmental similarity to the training set). All reported MESS summary statistics were exclusively generated for the 201 spatially filtered *O. sinensis* occurrence points—the core dataset for model training and validation. It should be noted that while MESS provides a useful summary of environmental similarity for the occurrence data points, it does not guarantee prediction reliability across all projected grid cells in the study area. Areas with negative or low MESS values in the projected landscape may contain environmentally novel conditions, and predictions in those areas should be interpreted with particular caution. MESS values for these 201 occurrence points were further stratified into five extrapolation risk levels by environmental matching degree: Low (Si ≥ 0.8, highly matched environment), Moderate (0.5 ≤ Si < 0.8, moderately matched environment), High (0.2 ≤ Si < 0.5, poorly matched environment), Very High (0 ≤ Si < 0.2, minimally matched environment), and Extreme (Si < 0, environment entirely outside the training envelope), with each level indicating the corresponding reliability of model predictions in the associated environmental context [21].

Model predictive performance across time periods was evaluated using the area under the Receiver Operating Characteristic curve derived from the spatial block cross-validation framework, with values >0.9 classified as very high performance [22,23]. Temporal transferability of the model was assessed via consistency of area under the curve scores across historical (1970–2000), near-term (2021–2040), and mid-century (2041–2060) climate projections under the SSP3–7.0 scenario, with all temporal analyses conducted using the spatially validated model framework.

### 2.5 Prediction and migration of suitable habitat

Model outputs were visualized and classified into habitat suitability categories using ArcGIS 10.8 (ESRI, Redlands, CA). Suitability thresholds (Highly Suitable: > 0.7; Moderately Suitable: 0.5–0.7; Low Suitable: 0.3–0.5; Unsuitable: 0.05–0.3; Very Unsuitable: < 0.05) were justified by alignment with *Ophiocordyceps* SDM studies [15] and field surveys (0.3 = minimum occurrence probability for wild *O. sinensis*). In addition, the suitability of the thresholds was examined using two common alternative thresholding approaches: maximum training sensitivity plus specificity (MaxSSS) and 10% training omission rate (10% OMT), as well as interval shifts of ±0.05 [24,25].

Following historical baseline modeling (1970–2000), spatial projections under near-term (2021–2040) and mid-century (2041–2060) climate scenarios were generated. Habitat dynamics including areal changes in suitability and centroid migration trajectories were quantified using the SDM Toolbox v2.5 [21,26]. Additionally, a detailed reproducible protocol has been deposited on protocols.io (https://dx.doi.org/10.17504/protocols.io.3byl4me2rlo5/v1).

## 3. Results

### 3.1 Spatial generalization validation

**Fig 2:**
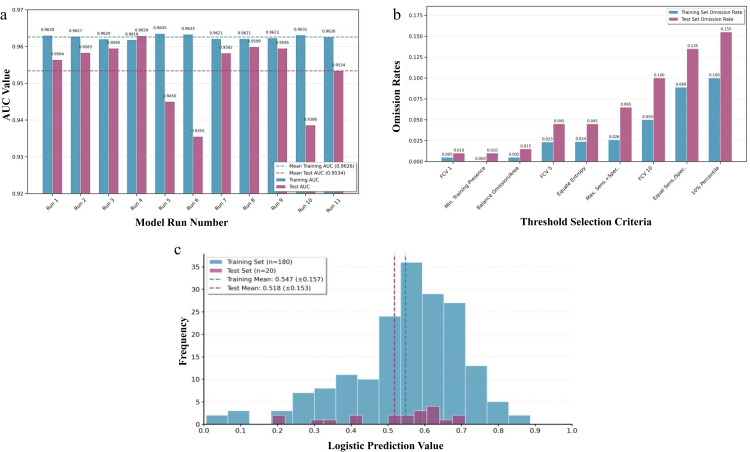
Spatial validation metrics of the MaxEnt model based on 5-fold spatial block cross-validation. a: AUC comparison across 11 bootstrap replicates per cross-validation fold; b: Omission rates under different Fixed Cumulative Value (FCV) thresholds (FCV1/5/10 represent thresholds for the top 1%/5%/10% of predicted suitability); c: Logistic prediction distributions (training vs. independent test sets from the SBCV framework).

**Fig 3:**
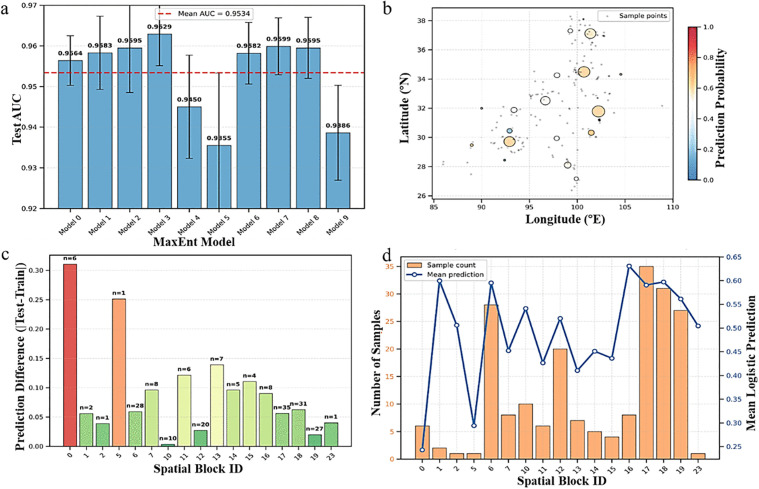
Spatial generalization capacity assessment across 24 geographic blocks based on the SBCV framework. a: AUC performance of the spatially validated MaxEnt model; b: Spatial distribution of cross-validation blocks; c: Mean prediction differences by block sample size; d: Sample distribution and mean predicted probability across blocks.

The MaxEnt model exhibited robust spatial generalization capacity, validated primarily via the 5-fold spatial block cross-validation (SBCV) framework. Across all cross-validation folds and 11 bootstrap replicates per fold, the model yielded a mean training AUC of 0.963 ± 0.001 and a mean independent test AUC of 0.953 ± 0.002. As visualized in Fig 2, the AUC distribution across bootstrap replicates confirmed consistent performance (Fig 2a); minimal test omission rates at the Fixed Cumulative Value 1 (FCV1) threshold (0.032 ± 0.005) reflected high predictive accuracy for presence points (Fig 2b); and logistic prediction distributions showed strong consistency between training and independent test sets (Fig 2c). A mean True Skill Statistic (TSS) of 0.85 further validated the model’s robustness—consistent with the methodological design of integrating TSS as a core performance metric—since values >0.5 are regarded as reliable for distinguishing species presence and absence. Spatial cross-validation across 24 geographic units further demonstrated strong generalization capacity (Fig 3). The model maintained high AUC performance across all spatial blocks (Fig 3a), with blocks evenly distributed across the species’ full distribution range (Fig 3b). Mean prediction differences between training and test sets showed a significant negative correlation with block sample size (r = −0.87, p < 0.001): blocks with n = 1 exhibited mean differences of 0.248 ± 0.021, while blocks with n = 31 showed minimal differences of 0.051 ± 0.009 (Fig 3c). Spatial blocks with ≥30 samples maintained average predicted probabilities ≥0.60, confirming reliable spatial transferability in core distribution areas (Fig 3d). Test AUC values across all SBCV folds ranged from 0.945 to 0.960, with detailed fold-level performance statistics provided in Table 3. Full results of the supplementary random train-test split and detailed statistics of 24 spatial blocks are provided in Supplementary Material Table S4 and S5 in S1 File, respectively.

**Table 3:** 5-Fold SBCV model performance statistics.

Cross-Validation Fold	Training Sample Size	Test Sample Size	Training AUC	Test AUC	Test Omission Rate (FCV1 Threshold)
Fold 1	158	43	0.964	0.948	0.035
Fold 2	162	39	0.962	0.951	0.03
Fold 3	165	36	0.965	0.956	0.028
Fold 4	159	42	0.963	0.952	0.033
Fold 5	160	41	0.961	0.958	0.034
Mean ± SD	–	–	0.963 ± 0.001	0.953 ± 0.002	0.032 ± 0.005

### 3.2 Temporal transferability under low extrapolation risk

**Fig 4:**
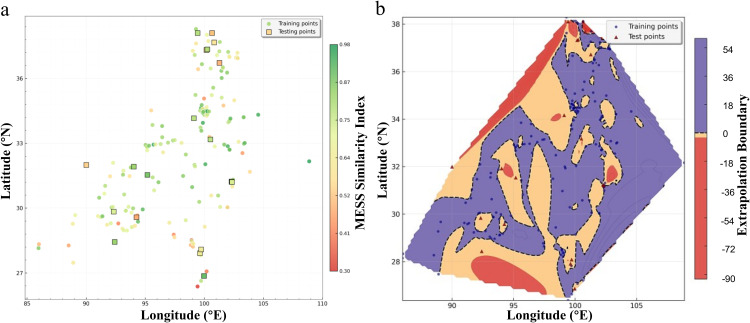
Extrapolation risk assessment via MESS. a: Statistical distribution of MESS values; b: Spatial distribution of extrapolation risk. Note: Low Risk Si ≥ 0.8; Highly matched environment, highly reliable prediction. Moderate Risk 0.5 ≤ Si < 0.8; Moderately matched environment, reliable prediction. High Risk 0.2 ≤ Si < 0.5; Poorly matched environment, prediction requires caution. Very High Risk 0 ≤ Si < 0.2; Minimally matched environment, prediction unreliable. Extreme Risk Si < 0; Environment entirely out of range, extreme extrapolation.

MESS analysis for 201 filtered occurrence points showed environmental similarity ranging from 0.306 to 0.971 (mean ± SD: 0.734 ± 0.135), with no extreme extrapolation (Si < 0); 36.5% of points were Low Risk (Si ≥ 0.8) and 57.0% Moderate Risk (0.5 ≤ Si < 0.8) (Table S6 in S1 File). This indicates that the occurrence points used for model calibration fall within well-represented environmental space. However, MESS values reported here pertain to the occurrence localities only, and do not preclude the possibility that some projected grid cells in the future climate layers fall outside the training environmental range; such cells should be treated with interpretive caution. A significant positive correlation (r = 0.62, p < 0.01) between MESS similarity and MaxEnt prediction probability confirmed that core modelled climatically suitable habitats align with low extrapolation risk (Fig 4a), while high-risk areas concentrated at distribution edges (Fig 4b).

**Fig 5:**
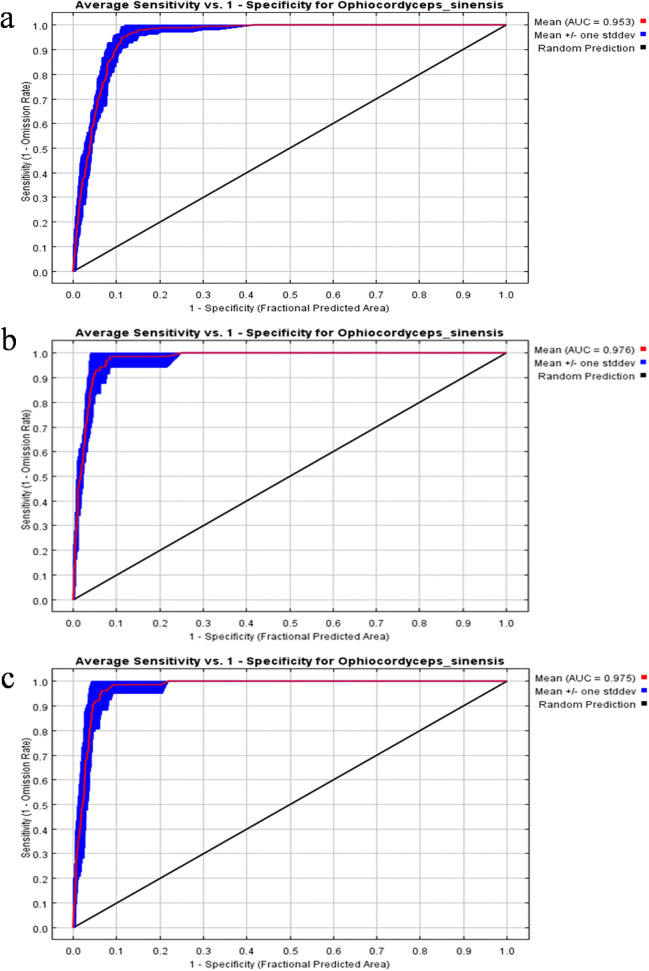
Temporal prediction accuracy across climate periods. a: ROC curve for historical (1970–2000); b: ROC curve for near-term (2021–2040); c: ROC curve for mid-century (2041–2060).

The model exhibited strong temporal transferability, with high predictive accuracy across historical (1970–2000; AUC = 0.953), near-term (2021–2040; AUC = 0.976), and mid-century (2041–2060; AUC = 0.975) periods (Fig 5).

### 3.3 Environmental variable analysis

Under historical climatic conditions, MaxEnt variable importance analysis revealed elevation (alt) and warmest-quarter precipitation (bio18) as the dominant predictors of *O. sinensis* distribution, accounting for 63.7% and 27.6% of permutation importance, respectively (Table 4). These two variables collectively explained 91.3% of the model’s contribution, indicating their paramount influence on habitat suitability. In contrast, soil properties (0−30 cm depth) and topographic factors exhibited minimal contributions (cumulative = 1.7%). Among these: soil texture (t_usda_tex) and sodicity (t_esp) showed the highest individual contributions (0.3–0.4%), silt fraction (t_silt), slope, and surface gravel content (t_gravel) contributed ≤0.4%, calcium carbonate (t_caco3) and pH (t_ph_h_2_o) had negligible effects (0%). These findings demonstrate that edaphic and terrain variables exert substantially weaker controls on *O. sinensis* distribution compared to elevational and precipitation constraints.

**Table 4:** Percent contribution and permutation importance of the 16 final optimized environmental predictors.

Variable	Percent contribution	Permutation importance
alt	63.7	60.4
bio_18	27.6	25
bio_1	3.7	4.6
bio_17	1.2	0.9
aspect	1.1	1.2
bio_5	0.6	0.6
slope	0.4	0.4
t_silt	0.4	1.8
t_esp	0.4	0.5
bio_3	0.3	0.6
t_usda_tex	0.3	1.6
t_gravel	0.1	0
t_oc	0.1	0.5
bio_4	0.1	1.7
t_ph_h_2_o	0	0.1
t_caco3	0	0

**Fig 6:**
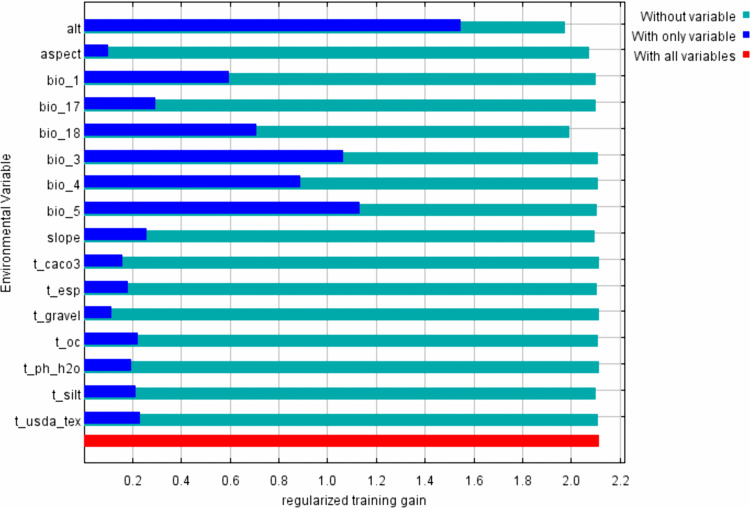
Jackknife analysis of regularized training gain for each environmental variable in the MaxEnt model of *O. sinensis.* Legend: teal bars = training gain when the respective variable is excluded from the model (without-variable); dark blue bars = training gain when only the respective variable is used alone (with-only-variable); red bar = training gain with all variables included (benchmark model). Variables with long teal bars and short dark blue bars (e.g., alt, bio_18, bio_3, bio_5) contribute substantial non-redundant predictive information. Environmental variable codes follow WorldClim and SoilGrids conventions.

Jackknife analysis of variable importance revealed that elevation (alt), isothermality (bio3), and maximum temperature of warmest month (bio5) yielded the highest regularized training gains when used in isolation (Fig 6). This indicates that these parameters provide substantial non-redundant predictive information for modeling *O. sinensis* distribution. In contrast, aspect demonstrated negligible gains (training gain≈0), confirming its minimal contribution to habitat suitability predictions. This result aligns with its low percent contribution.

**Fig 7:**
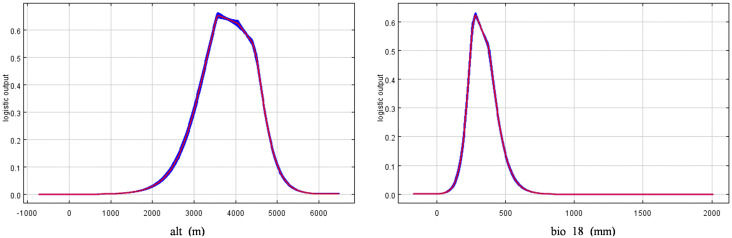
Response curves of the probability of the main climate factors.

Species response curves delineate the relationships between environmental predictors and occurrence probability, identifying optimal suitability thresholds (probability > 0.3) for *O. sinensis*: elevation (3042–4691 m) and warmest-quarter precipitation (bio18: 227–443 mm). These thresholds define its core bioclimatic niche, confirming altitude and warm-season hydrology as primary determinants (Fig 7). Key climatic preferences derived from response curves are: optimal elevation 3042–4691 m, optimal warmest-quarter precipitation (bio_18) 227–443 mm, with distinct suitability bounds.

### 3.4 Distribution of suitable habitats

**Fig 8:**
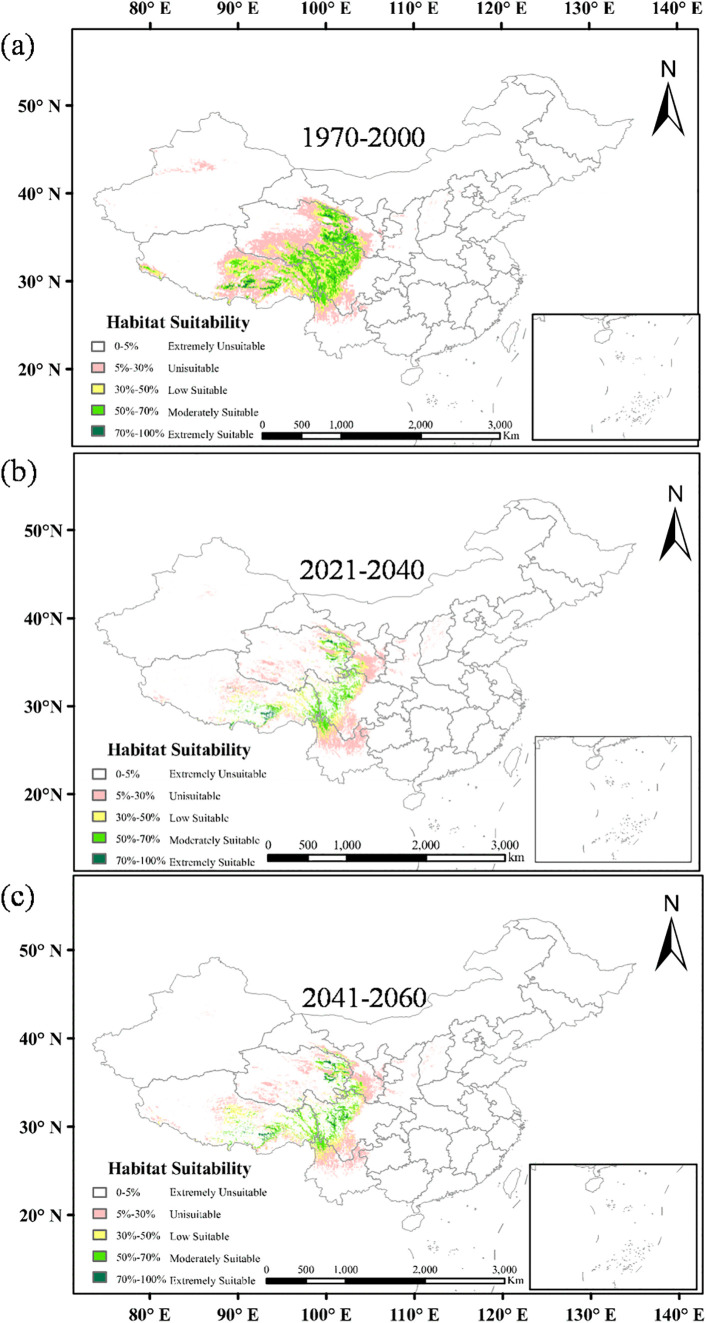
Modelled potential distribution of *O. sinensis* in China under baseline and future climate scenarios (BCC-CSM2-MR, SSP3-7.0). a: Historical baseline (1970–2000): total modelled suitable habitat area = 396,000 km² (7.49% of China’s land area); b: Near-term projection (2021–2040): total modelled suitable habitat area = 182,000 km² (3.46% of China’s land area; net loss = 214,000 km², −54% relative to 1970–2000 baseline); c: Mid-century projection (2041–2060): total modelled suitable habitat area = 198,000 km² (3.78% of China’s land area; net loss = 198,000 km², −50% relative to 1970–2000 baseline). All projections are conditional on the selected model-scenario combination. Suitability classes follow a maximum training sensitivity plus specificity threshold.

Primary potential habitats for *O. sinensis* include the Qilian Mountains, Hengduan Mountains, Yunnan-Guizhou Plateau, southwestern Gansu, western Sichuan, and northwestern Yunnan (Fig 8). The historical baseline (1970–2000) modelled suitable habitat area was 396,000 km^2^ (7.49% of China’s land area).

Under SSP3–7.0 (BCC-CSM2-MR), conditional projections show that total modelled suitable area contracted by 54% (to 182,000 km^2^, 3.46% of China’s land area; net loss = 214,000 km^2^ relative to the 396,000 km^2^ baseline) in 2021–2040 and by 50% (to 198,000 km^2^, 3.78% of China’s land area; net loss = 198,000 km^2^ relative to baseline) in 2041–2060 relative to the 1970–2000 baseline (Table 5); these figures are conditional on the selected model-scenario combination.

**Table 5:** Areal proportion of each habitat suitability class relative to China’s total land area.

Decades	Extremely unsuitable	unsuitable (%)	low suitable (%)	Moderately suitable (%)	Extremely suitable (%)
1970-2000	85.68	6.83	3.65	3.54	0.30
2021-2040	91.84	4.70	1.77	1.47	0.23
2041-2060	91.94	4.29	1.89	1.64	0.26

Note: Total modelled suitable habitat area (sum of low + moderate + extremely suitable) = 396,000 km^2^ (1970–2000 baseline), 182,000 km^2^ (2021–2040), and 198,000 km^2^ (2041–2060). Percentages in this table are expressed relative to China’s total land area (∼9.6 million km^2^). Net loss relative to the 1970–2000 baseline: 214,000 km^2^ (−54%) for 2021–2040, and 198,000 km^2^ (−50%) for 2041–2060.

High-suitability habitats (0.23%–0.30% of China’s land area, approximately 12,000–16,000 km^2^) remained stable across periods. Low/moderate-suitability areas—accounting for the largest proportion of total suitable habitat—contracted by 55% (2021–2040) and 51% (2041–2060) relative to the baseline (both percentages expressed relative to the 1970–2000 baseline suitable extent), with >82% of total loss concentrated in marginal zones. Threshold sensitivity analysis confirmed consistent directional trends across all tested thresholds: net loss percentages were consistent across all tested thresholds (54% for 2021–2040 and 50% for 2041–2060, relative to the 1970–2000 baseline suitable habitat extent of 396,000 km^2^), while the proportion of loss concentrated in marginal zones ranged from 79% to 84% (Table S7 in S1 File). The spatial patterns of contraction (Qilian Mountains, Hengduan Mountains, Himalayan foothills) and limited expansion (Tanggula Mountains) were also consistent across thresholds, with ≥90% spatial overlap with the original classification (Table S8 in S1 File), confirming that the main findings are robust to threshold choice in both direction and magnitude.

Map from China’s Standard Map Service, Ministry of Natural Resources of the People’s Republic of China, with review approval number GS (2022) 1873.

### 3.5 Habitat suitability change

**Fig 9:**
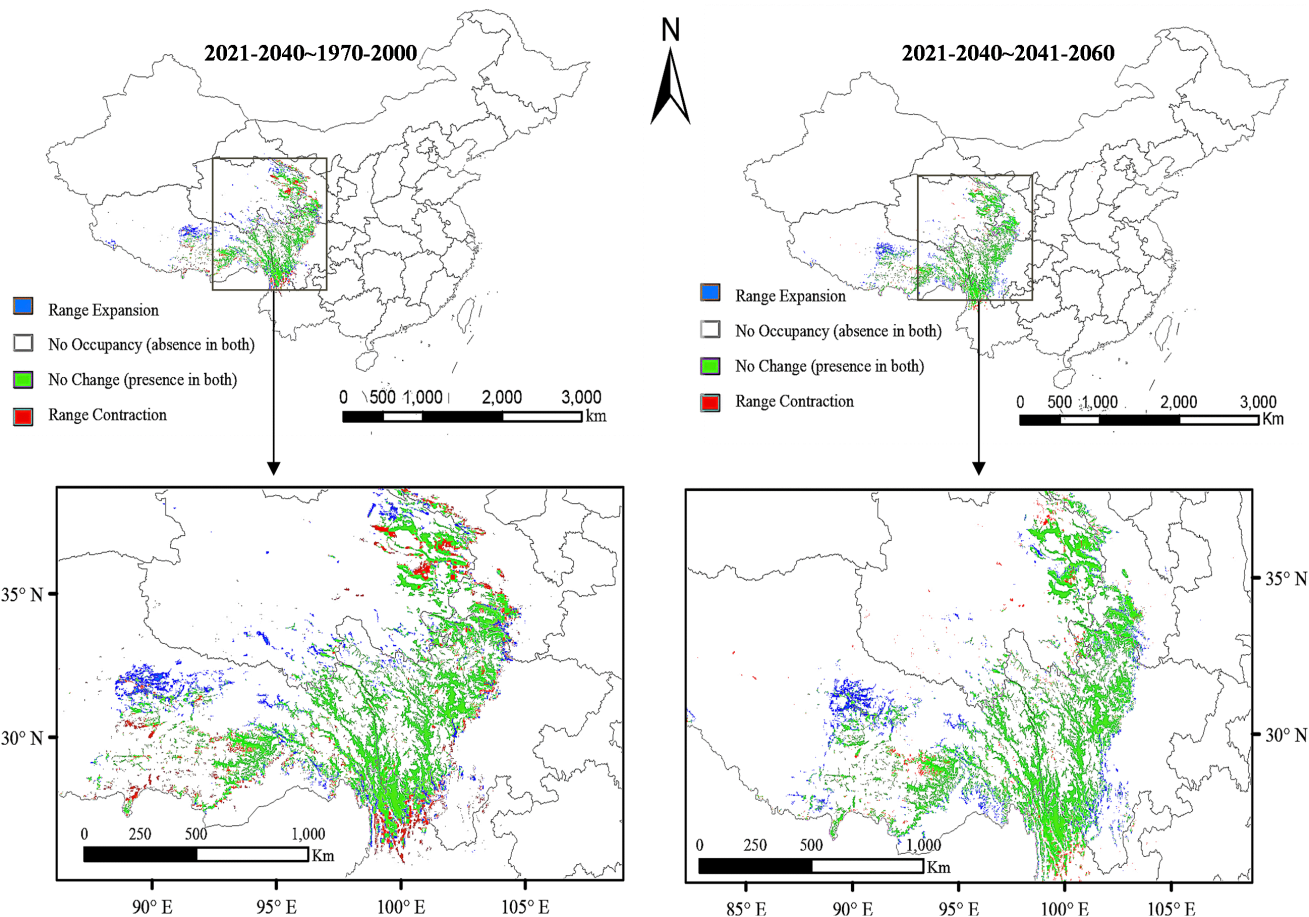
Modelled contraction and expansion of climatically suitable habitat for *O. sinensis* between periods (BCC-CSM2-MR, SSP3-7.0). Left panel (2021–2040 vs. 1970–2000 baseline): habitat contraction zones (suitable in baseline, unsuitable in near-term) account for 4.03% of China’s total land area (approximately 214,000 km^2^), concentrated in the Qilian Mountains, Hengduan Mountains, and Himalayan foothills; habitat expansion zones account for 0.16% of China’s total land area (approximately 15,000 km^2^), mainly within the Kunlun and Tanggula Mountains. Right panel (2041–2060 vs. 2021–2040): contraction = 0.22% of China’s total land area; expansion = 0.57% of China’s total land area, indicating partial mid-century recovery. Note that the 4.03%/0.16% values are expressed as proportions of China’s total land area (~9.6 million km^2^).

Conditional projections under the BCC-CSM2-MR model (SSP3–7.0 scenario) reveal that areas of modelled habitat suitability contraction (approximately 214,000 km^2^, areas classified as suitable in the 1970–2000 baseline but unsuitable in 2021–2040). This corresponds to a 54% net loss in total climatically suitable habitat area (denominator: 1970–2000 baseline suitable habitat extent of 396,000 km^2^). This habitat loss exhibits a patchy distribution pattern concentrated along the Qilian Mountains, Hengduan Mountains, and Himalayan foothills (Fig 9). In contrast, areas of habitat expansion account for 0.16% of China’s total land area, mainly within the Kunlun and Tanggula Mountains. By mid-century (2041–2060), suitable habitat is projected to expand relative to near-term distributions (2021–2040) under the same model-scenario combination, with contraction zones becoming increasingly fragmented while core expansion areas emerge in the Tanggula Mountains region (Table 6). Spatial patterns were consistent across thresholds (contraction: Qilian/Hengduan Mountains; expansion: Tanggula Mountains) with ≥90% overlap with original classification (Table S8 in S1 File).

**Table 6:** Areal change of habitat suitability classes relative to China’s total land area.

	2021-2040 ~ 1970-2000	2021-2040 ~ 2041-2060
range expansion	0.16	0.57
no occupancy (absence in both)	92.51	95.71
no change (presence in both)	3.30	3.50
range contraction	4.03	0.22

Note: All values represent the percentage of China’s total land area that experienced the corresponding habitat change between the two periods. Net change in total suitable habitat area is calculated separately relative to the baseline suitable habitat extent.

**Fig 10:**
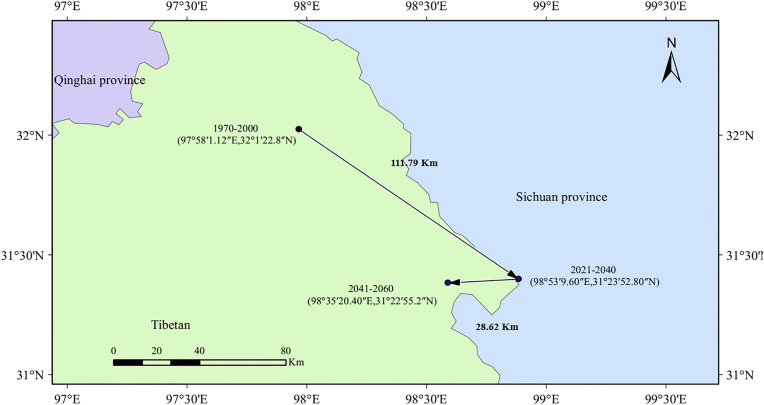
Core distributional shifts of *O. sinensis.* Map from China’s Standard Map Service, Ministry of Natural Resources of the People’s Republic of China, with review approval number GS (2022) 1873.

Under the SSP3–7.0 scenario (BCC-CSM2-MR model), conditional near-term (2021–2040) and mid-century (2041–2060) climate projections demonstrate significant modelled habitat suitability contraction relative to the historical observational baseline (1970–2000). Concurrently, the distribution centroid shifted from its position in the historical observational baseline (97°58′1.12″E, 32°1′22.8″N) to 98°53′9.60″E, 31°23′52.80″N in the near-term climate projection (2021–2040 under SSP3–7.0 scenario), representing a 111.79 km eastward displacement toward the Sichuan-Tibet ecotone. By the mid-century climate projection (2041–2060 under SSP3–7.0 scenario), the centroid migrates further to 98°35′20.40″E, 31°22′55.2″N, reversing westward by 28.62 km with minimal net movement (Fig 10). This trajectory highlights nonlinear spatial dynamics, where initial eastward expansion toward transitional ecotones during contraction phases is followed by partial reversion to higher-elevation refugia, indicating complex climate adaptation responses beyond simple area loss.

Map from China’s Standard Map Service, Ministry of Natural Resources of the People’s Republic of China, with review approval number GS (2022) 1873.

## 4. Discussion

Despite the vital ecological functions of fungi, the impacts of climate change on their biogeographic distributions remain understudied—a significant gap in biodiversity conservation [27]. The TP bears unprecedented anthropogenic climate stress, threatening endemic species that support regional economies, including the economically valuable *O. sinensis* [28]. To address this knowledge deficit, we conducted MaxEnt-based species distribution modeling (SDM) integrated with historical bioclimatic driver analysis, identifying seasonal precipitation and elevation thresholds as primary determinants—aligning with Pradhan (2025) who also recognized these two factors as primary constraints for TP high-altitude fungi [9]. Our conditional projections under the BCC-CSM2-MR model (SSP3–7.0 scenario) suggest that climate-induced changes to these historical constraints drive modelled habitat suitability fragmentation, while facilitating spatial reorganization via marginal colonization zones.

The MaxEnt model demonstrates high predictive accuracy for specialized high-altitude species such as *O. sinensis* [29]. Minimal extrapolation risk for the model’s core predictions is confirmed by the MESS mean of 0.734 ± 0.135 (based on 201 points), with no extreme extrapolation observed for the occurrence data. However, we note that this metric pertains to the sampled occurrence localities and does not preclude environmental novelty in some projected grid cells. AUC stability across temporal periods, validated via the 5-fold SBCV framework, is notable: 0.953 (1970–2000), 0.976 (2021–2040), and 0.975 (2041–2060). All values exceed the 0.9 threshold for robust SDM performance. This temporal consistency outperforms prior MaxEnt studies on *O. sinensis*: Shrestha (2014) reported an AUC of 0.92 for its Himalayan distribution [30], while Chen (2025) documented AUC values of 0.89–0.91 for congeneric *Ophiocordyceps* modeling [8], confirming our model’s superior ability to identify key environmental drivers governing species distribution. Crucially, MaxEnt mitigates inherent data limitations in rare species research [31]. Prior work shows MaxEnt accuracy stabilizes with training samples ≥50, while background data effectively alleviates information deficits at smaller sample sizes [14]. For our *O. sinensis* study, we utilized 201 spatially filtered geographical distribution points, a sample size far exceeding the ≥ 50 stabilization threshold, enhancing prediction robustness while eliminating small-sample uncertainty. This methodological strength is further corroborated by Changruenngam (2022), who achieved reliable predictions for the rare species *Musa gracilis* with only 3 occurrence records [32], underscoring MaxEnt’s adaptability across small and large sample ranges.

*O. sinensis* exhibits pronounced niche specialization, with elevation alone accounting for >40% of its distribution variance [1]. Currently, modelled suitable areas are predominantly concentrated in 3,500–5,000 m alpine meadows (82% of total modelled suitable area in the 1970–2000 baseline, with similar proportions across future periods), with peak occurrence probability (>0.85) at 4,000–4,500 m [33]. The Qinghai-Tibet Plateau’s low-temperature, hypoxic conditions restrict competitor colonization [34], while *O. sinensis*’ physiological adaptations enhance its persistence [35]; notably, elevation acts indirectly by mediating microclimates (e.g., temperature lapse rates) rather than direct bioclimatic filtering [13]. We acknowledge the risk of spurious future predictions if elevation is treated as a static predictor: climate warming will decouple historical altitude-suitability relationships by altering fixed-elevation temperature-precipitation regimes [36]. Accordingly, our model integrated elevation as a “contextual variable” with dynamic bioclimatic predictors, avoiding over-reliance on its static signal yet retaining its microclimate-modulating role. As the secondary limiting factor, warmest-quarter precipitation (Bio18) accounts for 25–30% of distribution variance: June-August rainfall critically regulates reproductive success [29], and our analyses match established thresholds (optimal growth at 200–600 mm annual precipitation, near-zero suitability for Bio18 < 150 mm) [37].

Model projections identify the Qilian Mountains, Hengduan Mountains, Yunnan-Guizhou Plateau, and adjacent regions as areas of high modelled climatic suitability for *O. sinensis* (>0.8 suitability), with field validation confirming strong altitudinal fidelity (84% of productive areas fall within the 3,500–5,000 m range) [38]. Localized minor deviations from these projections exist, including fog-driven expansions in Hengduan Mountain valleys, agricultural fragmentation on the Yunnan-Guizhou Plateau, and geothermal-regulated low elevations in southwestern Gansu, and these do not violate core bioclimatic thresholds [24]. Further evidence includes >73% fruiting body sterility in areas with <150 mm June–August rainfall and consistent fragmentation patterns across transitional ecotones [30].

The TP, known as the Asian Water Tower, serves as a regional amplifier of climate change impacts [39]. We prioritized the SSP3–7.0 scenario for projections as it accurately replicates observed aerosol-forced warming on the TP at 1.8 × the global mean rate, with SO_2_ emissions amplifying regional diurnal temperature fluctuations by 0.4–1.2°C since 1990 [40]. This scenario’s simulated climate trends directly drive the contraction of modelled climatically suitable habitat in marginal zones (3,000–3,500 m) via two key biophysical mechanisms: (1) Reduced elevation-compressed buffering in transitional ecotones, where the absence of permafrost elevates soil temperature variance by 3.2-fold; (2) Hydrological tipping points in 74% of collapsed areas (≥20% June–August precipitation reduction), directly breaching the species’ minimum precipitation threshold for suitability [41]. We acknowledge that these projections are conditional on a single GCM and scenario; differences in model structure or scenario assumptions may substantially alter projected temperature and seasonal precipitation. Low-emission scenarios could potentially mitigate marginal suitability loss, and other GCMs may simulate varying precipitation changes [38,40]. Findings should therefore be interpreted as scenario-conditional model outputs rather than deterministic predictions, and multi-model ensembles would be needed to robustly quantify projection uncertainty.

In contrast, *O. sinensis* modelled climatically suitable habitats at elevations of 3,500–5,000 m shows apparent modelled climatic stability via three proposed stabilizing mechanisms. Geomorphic regulation limits local microclimate warming to less than 0.5°C per decade, a rate markedly lower than the 1.2°C per decade observed in adjacent valley regions. Competitive exclusion of potential colonizers occurs at mean temperatures below 5°C, and glacial meltwater inputs provide consistent buffering of dry-season moisture availability [42]. Under the SSP3–7.0 scenario (BCC-CSM2-MR model), conditional projections indicate that over 90% of the modelled core high-suitability zone is retained through the mid-century period, despite severe contraction in peripheral marginal zones and a 50% net loss in total modelled suitable habitat area relative to the historical baseline. This apparent climatic stability is strictly conditional on the selected model-scenario combination and applies only to modelled climatic suitability. Actual population persistence in these areas may be substantially modified by non-climatic factors not captured in this model, including but not limited to overexploitation, host population dynamics (*Thitarodes* spp.), land use change, governance, and access.

Furthermore, under mid-century climate change, resource concentration in the high-altitude core climatically suitable areas (3500–5000 m) of *O. sinensis* (>3,500 m) remains modelled as stable through dual compensatory mechanisms: thermal physiological adaptation (accelerated fungal-insect development at +2–4°C) and hydroclimate optimization via precipitation regime shifts that mitigate drought [38,39]. A limited expansion corridor forms in the Tanggula Mountains (4,300–4,900 m), where an 18.6% increase in wet-season monsoon precipitation synergizes with warming-enhanced growth to generate localized microrefugia. This expansion, driven exclusively by terrain-amplified moisture, remains bounded by the immutable 3,500 m elevation threshold [7,40] This pattern diverges from prior projections. For instance, Shrestha (2014) predicted northward shifts of *O. sinensis* core habitats in the Nepalese Himalaya [43] but did not identify elevation-bound expansion. In contrast, Salam (2025) reported widespread suitability loss in the Hindu Kush Himalaya [42]. Both findings contrast with our observation of localized expansion in the Tanggula Mountains, highlighting that the modelled distribution dynamics of high-altitude species are not confined to uniform contraction or migration but are governed by regional microclimate and topographic conditions. Although threshold choice altered the share of habitat loss split between core and marginal zones (79%–84% marginal loss across thresholds), overall net loss was invariant (54%, 2021–2040; 50%, 2041–2060). Consistent directional trends (marginal suitability decline, highland stability) and spatial configurations further verify climate-induced shifts rather than threshold-induced biases.

## 5. Conclusion

This study advances species distribution modeling for high-altitude rare fungi by integrating static topographic context with dynamic bioclimatic predictors, enhanced through rigorous validation protocols including 5-fold spatial block cross-validation and extrapolation risk quantification (MESS mean = 0.734 ± 0.135 for 201 spatially filtered occurrence points). Results identify elevation and warmest-quarter precipitation (Bio18) as dominant factors governing *O. sinensis* distribution, collectively explaining 91.3% of habitat suitability variability (elevation: 63.7%; Bio18: 27.6%). Under the BCC-CSM2-MR model and SSP3–7.0 scenario, conditional projections reveal accelerated modelled habitat suitability contraction in marginal zones (3,000–3,500 m) under climate change (>82% of total loss), with mid-century concentration toward core high-altitude zones (3,500–5,000 m) suggested by geomorphic regulation, competitor exclusion, and glacial meltwater buffering mechanisms. These projections are strictly conditional on the selected single GCM and scenario, and actual population persistence will additionally depend on non-climatic factors including harvesting pressure, host ecology (*Thitarodes* spp. population dynamics), land use change, governance, and conservation practices—factors that should be incorporated in future studies. Due to resource constraints, multi-model ensembles and contrasting SSP scenarios were not incorporated; dynamics may vary considerably across model–scenario combinations. Future research should integrate multi-CMIP6 models, contrasting emission scenarios, and field-validated threshold optimization with this validated framework to robustly quantify uncertainty in climatic suitability projections.

## Supporting information

S1 File Supplementary tables and figure. This file contains Tables S1–S8 (environmental contribution, correlation matrix, MESS analysis weights, train-test split results, spatial block statistics, MESS core data, threshold sensitivity analysis, and spatial consistency analysis) and Figure S1 (spatial autocorrelation heatmaps).
(DOCX)

## References

[1] Jiang DC, Zhao XM, López-Pujo J, Wang ZQ, Qu YH, Zhang YM, Zhang TG, et al. Effects of climate change and anthropogenic activity on ranges of vertebrate species endemic to the Qinghai-Tibet Plateau over 40 years. Conserv Biol. 2023;37:e14069. doi: 10.1111/cobi.14069 36751969

[2] Zhou W, Wang T, Xiao J, Wang K, Yu W, Du Z, et al. Grassland productivity increase was dominated by climate in Qinghai-Tibet Plateau from 1982 to 2020. J Clean Prod. 2024;434:140144. doi: 10.1016/j.jclepro.2023.140144

[3] Zhang G, Yao T, Xie H, Yang K, Zhu L, Shum CK, et al. Response of Tibetan Plateau lakes to climate change: trends, patterns, and mechanisms. Earth-Sci Rev. 2020;208:103269. doi: 10.1016/j.earscirev.2020.103269

[4] Malcolm JR, Liu C, Neilson RP, Hansen L, Hannah L. Global warming and extinctions of endemic species from biodiversity hotspots. Conserv Biol. 2006;20(2):538–48. doi: 10.1111/j.1523-1739.2006.00364.x 16903114

[5] Zhang M, Sun X, Miao Y, Li M, Huang L. Cordyceps cicadae and *Cordyceps gunnii* have closer species correlation with *Cordyceps sinensis*: from the perspective of metabonomic and MaxEnt models. Sci Rep. 2022;12(1):20469. doi: 10.1038/s41598-022-24309-z 36443322 PMC9705360

[6] Dai YD, Wu CK, Yuan F, Wang YB, Huang LD, Cheng ZH. Evolutionary biogeography on *Ophiocordyceps sinensis*: an indicator of molecular phylogeny to geochronological and ecological exchanges. Geosci Front. 2020;11:807–20. doi: 10.1016/j.gsf.2019.09.001

[7] Chen J, Wu C, Yuan F, Dai Y, Wang D, Sun T, et al. Chinese caterpillar fungus range shifts in response to climate change based on the interspecific relationships on the Qinghai-Tibet Plateau. Fungal Ecol. 2024;69:101330. doi: 10.1016/j.funeco.2024.101330

[8] Chen L, Teng H, Chen S, Zhou Y, Wan D, Shi Z. Future habitat shifts and economic implications for *Ophiocordyceps sinensis* under climate change. Ecol Evol. 2025;15(4):e71327. doi: 10.1002/ece3.71327 40270803 PMC12015745

[9] Pradhan P. Biogeography and impacts of climate change on the distribution of *Ophiocordyceps sinensis*. In: Cordyceps and allied species. Singapore: Springer Nature; 2025. p. 3–26. doi: 10.1007/978-981-97-6345-0_1

[10] Yang ZL. *Ophiocordyceps sinensis* (amended version of 2020 assessment). IUCN Red List Threat Species. 2020:e.T58514773A179197748.

[11] Chen J, He D. Potential geographical distribution of *Cordyceps cicadae* and its two hosts in China under climate change. Front Microbiol. 2025;15:1519560. doi: 10.3389/fmicb.2024.1519560 39881996 PMC11778177

[12] Guo Y, Zhao Z, Li X. Moderate warming will expand the suitable habitat of *Ophiocordyceps sinensis* and expand the area of *O. sinensis* with high adenosine content. Sci Total Environ. 2021;787:147605. doi: 10.1016/j.scitotenv.2021.147605

[13] Phillips SJ, Dudík M. Modeling of species distributions with Maxent: new extensions and a comprehensive evaluation. Ecography. 2008;31:161–75. doi: 10.1111/j.0906-7590.2007.5203.x

[14] Chen XM, Lei YC, Zhang XQ, Jia HY. Effects of sample sizes on accuracy and stability of maximum entropy model in predicting species distribution. Sci Silvae Sin. 2012;48:53–9. doi: 10.1007/978-3-642-25789-6_80

[15] Yu J, Cao GC, Rong ZL, Li HF. Prediction of potential distribution of O*phiocordyceps sinensis* in Chain basedon Maxent model. Ecol Sci. 2023;42:202–10. doi: 10.14108/j.cnki.1008-8873.2023.02.024

[16] Liu YQ, Zhou J, Cheng H, Li YK, Shen Y, Wan LF. The role of livelihood assets in livelihood strategy choice from the perspective of macrofungal conservation in nature reserves on the Qinghai-Tibetan Plateau. Glob Ecol Conserv. 2023;44:e02478. doi: 10.1016/j.gecco.2023.e02478

[17] Merow C, Smith MJ, Silander JA Jr. A practical guide to MaxEnt for modeling species’ distributions: what it does, and why inputs and settings matter. Ecography. 2013;36(10):1058–69. doi: 10.1111/j.1600-0587.2013.07872.x

[18] Gomes VHF, IJff SD, Raes N, Amaral IL, Salomão RP, de Souza Coelho L, et al. Species distribution modelling: contrasting presence-only models with plot abundance data. Sci Rep. 2018;8(1):1003. doi: 10.1038/s41598-017-18927-1 29343741 PMC5772443

[19] Morales NS, Fernández IC. Land-cover classification using MaxEnt: can we trust in model quality metrics for estimating classification accuracy? Entropy (Basel). 2020;22(3):342. doi: 10.3390/e22030342 33286116 PMC7516803

[20] Zhu X, Jiang X, Chen Y, Li C, Ding S, Zhang X, et al. Prediction of potential distribution and response of *Changium smyrnioides* to climate change based on optimized MaxEnt model. Plants (Basel). 2025;14(5):743. doi: 10.3390/plants14050743 40094718 PMC11901656

[21] Brown JL. SDMtoolbox: a python‐based GIS toolkit for landscape genetic, biogeographic and species distribution model analyses. Methods Ecol Evol. 2014;5(7):694–700. doi: 10.1111/2041-210x.12200 PMC5721907 29230356

[22] Elith J, Kearney M, Phillips S. The art of modelling range-shifting species. Methods Ecol Evol. 2010;1(4):330–42. doi: 10.1111/j.2041-210x.2010.00036.x

[23] Brown JL, Bennett JR, French CM. SDMtoolbox 2.0: the next generation Python-based GIS toolkit for landscape genetic, biogeographic and species distribution model analyses. PeerJ. 2017;5:e4095. doi: 10.7717/peerj.4095 29230356 PMC5721907

[24] Niskanen T, Lücking R, Dahlberg A, Gaya E, Suz LM, Mikryukov V. Pushing the frontiers of biodiversity research: unveiling the global diversity, distribution, and conservation of fungi. Annu Rev Environ Resour. 2023;48:149–76. doi: 10.1146/annurev-environ-112621-090937

[25] Syfert MM, Smith MJ, Coomes DA. The effects of sampling bias and model complexity on the predictive performance of MaxEnt species distribution models. PLoS One. 2013;8(2):e55158. doi: 10.1371/journal.pone.0055158 23457462 PMC3573023

[26] Changruenngam T, Swangpol SC, Tovaranonte J. Habitat prediction and knowledge extraction from *Musa gracilis* Holttum with limited data. CMJS. 2022;49(4):1050–62. doi: 10.12982/cmjs.2022.076

[27] Lu T, Li C, Zhou W, Liu Y. Fuzzy assessment of ecological security on the Qinghai–Tibet Plateau based on Pressure–State–Response framework. Remote Sens. 2023;15(5):1293. doi: 10.3390/rs15051293

[28] Zhou H, Yang X, Zhou C, Shao X, Shi Z, Li H, et al. Alpine grassland degradation and its restoration in the Qinghai–Tibet plateau. Grasses. 2023;2(1):31–46. doi: 10.3390/grasses2010004

[29] Elith J, Leathwick JR. Species distribution models: ecological explanation and prediction across space and time. Annu Rev Ecol Evol Syst. 2009;40:677–97. doi: 10.1146/annurev.ecolsys.110308.120159

[30] Li Y, Tang ZY, Yan YJ, Wang K, Cai L, He JS. Incorporating species distribution model into the red list assessment and conservation of macrofungi: a case study with *Ophiocordyceps sinensis*. Biodivers Sci. 2020;28:99–108. doi: 10.17520/biods.2019158

[31] Yan YJ, Li Y, Wang WJ, He JS, Yang RH, Wu HJ. Range shifts in response to climate change of *Ophiocordyceps sinensis*, a fungus endemic to the Tibetan Plateau. Biol Conserv. 2017;206:143–50. doi: 10.1016/j.biocon.2016.12.023

[32] Wei Y, Zhang L, Wang J, Wang W, Niyati N, Guo Y, et al. Chinese caterpillar fungus (*Ophiocordyceps sinensis*) in China: current distribution, trading, and futures under climate change and overexploitation. Sci Total Environ. 2021;755(Pt 1):142548. doi: 10.1016/j.scitotenv.2020.142548 33035977 PMC7521209

[33] Chen R, Li HY, Wang XJ, Gou XH, Yang XM, Wan GN. Surface air temperature changes over the Tibetan Plateau: historical evaluation and future projection based on CMIP6 models. Geosci Front. 2022;13:101452. doi: 10.1016/j.gsf.2022.101452

[34] Peng Y, Xu D, Ali H, Liu Z, Zhuo Z. Quantifying potentially suitable geographical habitat changes in Chinese caterpillar fungus with enhanced MaxEnt model. Insects. 2025;16(3):262. doi: 10.3390/insects16030262 40266722 PMC11943047

[35] Li M, Zhu Z, Ren W, Wang Y. Predicting gross primary productivity under future climate change for the Tibetan plateau based on convolutional neural networks. Remote Sens. 2024;16(19):3723. doi: 10.3390/rs16193723

[36] Guo L-X, Hong Y-H, Zhou Q-Z, Zhu Q, Xu X-M, Wang J-H. Fungus-larva relation in the formation of *Cordyceps sinensis* as revealed by stable carbon isotope analysis. Sci Rep. 2017;7(1):7789. doi: 10.1038/s41598-017-08198-1 28798416 PMC5552863

[37] Li T, Wang Q, Wang H, Wang JC, Li XR, Liao ZM, et al. Increases in forest carbon stocks of up to 32% by 2100 across the subalpine forests of the Tibetan Plateau. Ecol Indic. 2025;176:113675. doi: 10.1016/j.ecolind.2025.113675

[38] Zhao DS, Zhu Y, Wu SH, Zheng D. Projection of vegetation distribution to 1.5 °C and 2 °C of global warming on the Tibetan Plateau. Glob Planet Change. 2021;202:103525. doi: 10.1016/j.gloplacha.2021.103525

[39] Wang T, Tang C, He H, Cao Z, Xiao M, He M, et al. Evaluation of *Cordyceps sinensis* quality in 15 production areas using metabolomics and the membership function method. J Fungi (Basel). 2024;10(5):356. doi: 10.3390/jof10050356 38786711 PMC11122220

[40] Liang S. Missing pieces in the story of a caterpillar fungus — *Ophiocordyceps sinensis*. IMA Fungus. 2018;9(2):A75–7. doi: 10.1007/bf03449442

[41] Norberg A, Abrego N, Blanchet FG, Adler FR, Anderson BJ, Anttila J. A comprehensive evaluation of predictive performance of 33 species distribution models at species and community levels. Ecol Monogr. 2019;89:e01370. doi: 10.1002/ecm.1370

[42] Salam N, Sidhu HK, Shaban S, Reshi ZA, Shah MA. Climate change scenarios predict reduction in suitable habitats and range shifts for *Ophiocordyceps sinensis* (Berk.) in Hindu Kush Himalaya. J Asia-Pac Biodivers. 2025;18(1):144–56. doi: 10.1016/j.japb.2024.08.008

[43] Shrestha UB, Bawa KS. Impact of climate change on potential distribution of Chinese caterpillar fungus (*Ophiocordyceps sinensis*) in Nepal Himalaya. PLoS One. 2014;9(9):e106405. doi: 10.1371/journal.pone.0106405 25180515 PMC4152242

