# Supplementary material for: Modelled climatic suitability contraction and high-altitude persistence: Projected mid-century distribution dynamics of Ophiocordyceps sinensis across the Tibetan Plateau under a selected climate scenario
Source: PLoS One. 2026 Jul 21;21(7):e0354200. doi: 10.1371/journal.pone.0354200 (PMC13387540; doi:10.1371/journal.pone.0354200)
Supplement: S1 File — This file contains Tables S1–S8 (environmental contribution, correlation matrix, MESS analysis weights, train-test split results, spatial block statistics, MESS core data, threshold sensitivity analysis, and spatial consistency analysis) and Figure S1 (spatial autocorrelation heatmaps). (DOCX) [file pone.0354200.s001.docx]

Table S1 Percentage of environmental contribution of prebuilt models

| Variable | Percent contribution | Permutation importance |
| --- | --- | --- |
| alt | 71.1 | 69.5 |
| bio_18 | 16.1 | 9 |
| bio_1 | 3.5 | 2.8 |
| slope | 2.7 | 1 |
| aspect | 1.7 | 0.8 |
| t_caco3 | 1.4 | 2.5 |
| t_ph_h2o | 1 | 0.5 |
| t_oc | 0.5 | 0.3 |
| bio_4 | 0.3 | 6.8 |
| bio_14 | 0.3 | 0 |
| bio_5 | 0.2 | 0.1 |
| t_esp | 0.2 | 0.3 |
| t_silt | 0.2 | 0.4 |
| bio_17 | 0.1 | 1.1 |
| bio_2 | 0.1 | 0.5 |
| bio_12 | 0.1 | 0.6 |
| bio_11 | 0.1 | 0.3 |
| bio_16 | 0.1 | 2.2 |
| bio_19 | 0.1 | 0.1 |
| t_usda_tex | 0.1 | 0.2 |
| bio_3 | 0.1 | 0.2 |
| t_gravel | 0.1 | 0.1 |
| t_caso4 | 0 | 0 |
| bio_15 | 0 | 0.3 |
| t_sand | 0 | 0 |
| t_clay | 0 | 0.1 |
| bio_6 | 0 | 0 |
| t_teb | 0 | 0.1 |
| bio_8 | 0 | 0 |
| t_bs | 0 | 0 |
| bio_13 | 0 | 0 |
| t_ece | 0 | 0 |
| t_cec_soil | 0 | 0 |
| bio_9 | 0 | 0 |
| bio_10 | 0 | 0 |
| t_ref_bulk | 0 | 0 |
| bio_7 | 0 | 0 |
| t_cec_clay | 0 | 0 |

Table S2 Correlation matrix of environmental factors of the *Ophiocordyceps sinensis*

|  | t_usda_tex | alt | aspect | bio_1 | bio_2 | bio_3 | bio_4 | bio_5 | bio_11 | bio_12 | bio_14 | bio_16 | bio_17 | bio_18 | bio_19 | slope | t_caco3 | t_esp | t_gravel | t_oc | t_ph_h2o | t_silt |
| --- | --- | --- | --- | --- | --- | --- | --- | --- | --- | --- | --- | --- | --- | --- | --- | --- | --- | --- | --- | --- | --- | --- |
| t_usda_tex | 1 | 0.299347 | -0.0205 | -0.36699 | 0.437771 | 0.12231 | 0.184894 | -0.22377 | -0.36709 | -0.45918 | -0.4388 | -0.43858 | -0.43919 | -0.41286 | -0.41742 | 0.017337 | -0.03403 | -0.08221 | 0.202797 | -0.1966 | 0.021979 | -0.30657 |
| alt | 0.299347 | 1 | -0.01474 | -0.70044 | 0.413955 | 0.689078 | -0.37289 | -0.88939 | -0.38564 | -0.37603 | -0.3732 | -0.37309 | -0.36701 | -0.35828 | -0.31936 | 0.271861 | -0.20979 | -0.12292 | 0.303706 | 0.168455 | -0.2372 | 0.158572 |
| aspect | -0.0205 | -0.01474 | 1 | -0.02307 | -0.01219 | -0.04431 | 0.048592 | 0.001794 | -0.03925 | 0.023251 | 0.033917 | 0.020609 | 0.034333 | 0.012093 | 0.04139 | 0.006078 | 0.004768 | -0.01687 | -0.02287 | 0.015896 | -0.01323 | 0.011403 |
| bio_1 | -0.36699 | -0.70044 | -0.02307 | 1 | -0.60418 | -0.14399 | -0.31677 | 0.803155 | 0.913203 | 0.643653 | 0.623269 | 0.585736 | 0.621499 | 0.547505 | 0.589986 | -0.13755 | 0.180525 | 0.086593 | -0.031 | -0.13042 | 0.005615 | -0.22079 |
| bio_2 | 0.437771 | 0.413955 | -0.01219 | -0.60418 | 1 | 0.282582 | 0.46703 | -0.23635 | -0.67385 | -0.84834 | -0.75819 | -0.80825 | -0.75117 | -0.78119 | -0.71048 | 0.025219 | 0.111843 | 0.139979 | -0.07918 | -0.07454 | 0.179103 | -0.09381 |
| bio_3 | 0.12231 | 0.689078 | -0.04431 | -0.14399 | 0.282582 | 1 | -0.67508 | -0.50957 | 0.171317 | -0.04378 | -0.16998 | -0.02738 | -0.15793 | -0.00825 | -0.11204 | 0.224028 | -0.1462 | -0.08213 | 0.289534 | 0.104571 | -0.26867 | -0.00419 |
| bio_4 | 0.184894 | -0.37289 | 0.048592 | -0.31677 | 0.46703 | -0.67508 | 1 | 0.292342 | -0.67492 | -0.55837 | -0.43606 | -0.51612 | -0.44033 | -0.49913 | -0.4506 | -0.20358 | 0.175133 | 0.164313 | -0.36063 | -0.13717 | 0.367256 | -0.05507 |
| bio_5 | -0.22377 | -0.88939 | 0.001794 | 0.803155 | -0.23635 | -0.50957 | 0.292342 | 1 | 0.50008 | 0.235509 | 0.323214 | 0.189151 | 0.319629 | 0.154209 | 0.285937 | -0.24098 | 0.327765 | 0.213583 | -0.24115 | -0.23713 | 0.242699 | -0.28852 |
| bio_11 | -0.36709 | -0.38564 | -0.03925 | 0.913203 | -0.67385 | 0.171317 | -0.67492 | 0.50008 | 1 | 0.740548 | 0.677525 | 0.675503 | 0.678055 | 0.637188 | 0.657612 | -0.02183 | 0.068119 | -0.00289 | 0.131432 | -0.04403 | -0.15142 | -0.14571 |
| bio_12 | -0.45918 | -0.37603 | 0.023251 | 0.643653 | -0.84834 | -0.04378 | -0.55837 | 0.235509 | 0.740548 | 1 | 0.855706 | 0.973453 | 0.858633 | 0.938004 | 0.837503 | -0.08114 | -0.20088 | -0.1831 | 0.091724 | 0.113961 | -0.33132 | 0.024338 |
| bio_14 | -0.4388 | -0.3732 | 0.033917 | 0.623269 | -0.75819 | -0.16998 | -0.43606 | 0.323214 | 0.677525 | 0.855706 | 1 | 0.747615 | 0.993552 | 0.659374 | 0.971822 | -0.08812 | -0.10883 | -0.11552 | 0.106176 | 0.069194 | -0.30028 | -0.01904 |
| bio_16 | -0.43858 | -0.37309 | 0.020609 | 0.585736 | -0.80825 | -0.02738 | -0.51612 | 0.189151 | 0.675503 | 0.973453 | 0.747615 | 1 | 0.749622 | 0.983687 | 0.72791 | -0.10362 | -0.22194 | -0.19541 | 0.067512 | 0.121759 | -0.3063 | 0.049886 |
| bio_17 | -0.43919 | -0.36701 | 0.034333 | 0.621499 | -0.75117 | -0.15793 | -0.44033 | 0.319629 | 0.678055 | 0.858633 | 0.993552 | 0.749622 | 1 | 0.658411 | 0.980256 | -0.08649 | -0.11224 | -0.11939 | 0.10424 | 0.073366 | -0.30529 | -0.02005 |
| bio_18 | -0.41286 | -0.35828 | 0.012093 | 0.547505 | -0.78119 | -0.00825 | -0.49913 | 0.154209 | 0.637188 | 0.938004 | 0.659374 | 0.983687 | 0.658411 | 1 | 0.628016 | -0.10162 | -0.22642 | -0.19555 | 0.063052 | 0.124334 | -0.28158 | 0.066836 |
| bio_19 | -0.41742 | -0.31936 | 0.04139 | 0.589986 | -0.71048 | -0.11204 | -0.4506 | 0.285937 | 0.657612 | 0.837503 | 0.971822 | 0.72791 | 0.980256 | 0.628016 | 1 | -0.07194 | -0.12087 | -0.1199 | 0.122853 | 0.076309 | -0.32292 | -0.02701 |
| slope | 0.017337 | 0.271861 | 0.006078 | -0.13755 | 0.025219 | 0.224028 | -0.20358 | -0.24098 | -0.02183 | -0.08114 | -0.08812 | -0.10362 | -0.08649 | -0.10162 | -0.07194 | 1 | 0.074545 | -0.01912 | 0.092685 | 0.017305 | 0.031328 | 0.08703 |
| t_caco3 | -0.03403 | -0.20979 | 0.004768 | 0.180525 | 0.111843 | -0.1462 | 0.175133 | 0.327765 | 0.068119 | -0.20088 | -0.10883 | -0.22194 | -0.11224 | -0.22642 | -0.12087 | 0.074545 | 1 | 0.35947 | -0.16054 | -0.18758 | 0.70246 | 0.264023 |
| t_esp | -0.08221 | -0.12292 | -0.01687 | 0.086593 | 0.139979 | -0.08213 | 0.164313 | 0.213583 | -0.00289 | -0.1831 | -0.11552 | -0.19541 | -0.11939 | -0.19555 | -0.1199 | -0.01912 | 0.35947 | 1 | -0.10573 | -0.13038 | 0.343233 | 0.029816 |
| t_gravel | 0.202797 | 0.303706 | -0.02287 | -0.031 | -0.07918 | 0.289534 | -0.36063 | -0.24115 | 0.131432 | 0.091724 | 0.106176 | 0.067512 | 0.10424 | 0.063052 | 0.122853 | 0.092685 | -0.16054 | -0.10573 | 1 | 0.127054 | -0.26537 | -0.08066 |
| t_oc | -0.1966 | 0.168455 | 0.015896 | -0.13042 | -0.07454 | 0.104571 | -0.13717 | -0.23713 | -0.04403 | 0.113961 | 0.069194 | 0.121759 | 0.073366 | 0.124334 | 0.076309 | 0.017305 | -0.18758 | -0.13038 | 0.127054 | 1 | -0.20498 | 0.197804 |
| t_ph_h2o | 0.021979 | -0.2372 | -0.01323 | 0.005615 | 0.179103 | -0.26867 | 0.367256 | 0.242699 | -0.15142 | -0.33132 | -0.30028 | -0.3063 | -0.30529 | -0.28158 | -0.32292 | 0.031328 | 0.70246 | 0.343233 | -0.26537 | -0.20498 | 1 | 0.264528 |
| t_silt | -0.30657 | 0.158572 | 0.011403 | -0.22079 | -0.09381 | -0.00419 | -0.05507 | -0.28852 | -0.14571 | 0.024338 | -0.01904 | 0.049886 | -0.02005 | 0.066836 | -0.02701 | 0.08703 | 0.264023 | 0.029816 | -0.08066 | 0.197804 | 0.264528 | 1 |

Table S3. Environmental variable contribution and MESS analysis weight

| Environmental Variable | Variable Classification | Percent Contribution to MaxEnt Model | Permutation Importance | Weight for MESS Analysis |
| --- | --- | --- | --- | --- |
| alt (Elevation) | Terrain factor | 63.7 | 60.4 | 0.633 |
| bio_18 (Precipitation of warmest quarter) | Bioclimatic variable | 27.6 | 25 | 0.274 |
| bio_1 (Annual mean temperature) | Bioclimatic variable | 3.7 | 4.6 | 0.038 |
| bio_17 (Precipitation of driest quarter) | Bioclimatic variable | 1.2 | 0.9 | 0.013 |
| aspect (Aspect) | Terrain factor | 1.1 | 1.2 | 0.013 |
| bio_5 (Max temperature of warmest month) | Bioclimatic variable | 0.6 | 0.6 | 0.005 |
| slope (Slope) | Terrain factor | 0.4 | 0.4 | 0.005 |
| t_silt (Topsoil silt fraction) | Soil variable | 0.4 | 1.8 | 0.004 |
| bio_3 (Isothermality) | Bioclimatic variable | 0.3 | 0.6 | 0.004 |
| t_esp (Topsoil sodicity (ESP)) | Soil variable | 0.4 | 0.5 | 0.003 |
| t_usda_tex (USDA soil texture classification) | Soil variable | 0.3 | 1.6 | 0.003 |
| t_gravel (Topsoil gravel content) | Soil variable | 0.1 | 0 | 0.002 |
| t_oc (Topsoil organic carbon) | Soil variable | 0.1 | 0.5 | 0.002 |
| bio_4 (Temperature seasonality) | Bioclimatic variable | 0.1 | 1.7 | 0.001 |
| t_ph_h2o (Topsoil pH (H2O)) | Soil variable | 0 | 0.1 | 0 |
| t_caco3 (Topsoil calcium carbonate) | Soil variable | 0 | 0 | 0 |

Table S4. Supplementary results of 75%/25% random Train-Test Split validation

| Iteration Number | Training AUC | Test AUC | Omission Rate (FCV1 Threshold) | Mean Prediction Probability of Test Set |
| --- | --- | --- | --- | --- |
| 1 | 0.964 | 0.955 | 0.031 | 0.612 |
| 2 | 0.963 | 0.952 | 0.033 | 0.608 |
| 3 | 0.962 | 0.951 | 0.034 | 0.605 |
| 4 | 0.964 | 0.954 | 0.032 | 0.61 |
| 5 | 0.963 | 0.953 | 0.032 | 0.609 |
| 6 | 0.963 | 0.953 | 0.032 | 0.609 |
| 7 | 0.964 | 0.954 | 0.031 | 0.611 |
| 8 | 0.962 | 0.952 | 0.033 | 0.607 |
| 9 | 0.963 | 0.953 | 0.032 | 0.609 |
| 10 | 0.963 | 0.953 | 0.032 | 0.609 |
| 11 | 0.963 | 0.953 | 0.032 | 0.609 |
| Mean ± SD | 0.963 ± 0.001 | 0.953 ± 0.002 | 0.032 ± 0.001 |  |

Table S5. Detailed statistics of 24 spatial cross-validation blocks

| Spatial Block ID | Number of Samples in Block | Mean Prediction Probability | Mean Prediction Difference between Training and Test Sets |
| --- | --- | --- | --- |
| 1 | 1 | 0.321 | 0.248 ± 0.021 |
| 2 | 1 | 0.335 | 0.242 ± 0.018 |
| 3 | 2 | 0.387 | 0.215 ± 0.016 |
| 4 | 2 | 0.392 | 0.209 ± 0.015 |
| 5 | 4 | 0.423 | 0.187 ± 0.012 |
| 6 | 5 | 0.456 | 0.162 ± 0.011 |
| 7 | 6 | 0.489 | 0.145 ± 0.010 |
| 8 | 7 | 0.512 | 0.128 ± 0.009 |
| 9 | 8 | 0.534 | 0.112 ± 0.008 |
| 10 | 10 | 0.557 | 0.098 ± 0.007 |
| 11 | 10 | 0.562 | 0.095 ± 0.007 |
| 12 | 12 | 0.585 | 0.087 ± 0.006 |
| 13 | 15 | 0.598 | 0.079 ± 0.006 |
| 14 | 18 | 0.521 | 0.072 ± 0.005 |
| 15 | 20 | 0.535 | 0.068 ± 0.005 |
| 16 | 20 | 0.542 | 0.065 ± 0.005 |
| 17 | 23 | 0.558 | 0.061 ± 0.004 |
| 18 | 25 | 0.571 | 0.058 ± 0.004 |
| 19 | 28 | 0.584 | 0.055 ± 0.003 |
| 20 | 31 | 0.602 | 0.051 ± 0.009 |
| 21 | 31 | 0.615 | 0.049 ± 0.008 |
| 22 | 31 | 0.621 | 0.047 ± 0.008 |
| 23 | 31 | 0.628 | 0.045 ± 0.007 |
| 24 | 31 | 0.635 | 0.042 ± 0.007 |

Table S6 MESS core statistical data

| Statistical Metric | Training Set (n=180) | Test Set (n=20) | Full Dataset (n=200) |
| --- | --- | --- | --- |
| MESS Similarity Mean | 0.737 | 0.709 | 0.734 |
| MESS Similarity Standard Deviation (SD) | 0.132 | 0.151 | 0.135 |
| MESS Similarity Range | 0.306-0.971 | 0.321-0.958 | 0.306-0.971 |
| Number of Low-Risk Samples (≥0.8) | 67 | 6 | 73 |
| Number of Medium-Risk Samples (0.5 - 0.8) | 102 | 12 | 114 |
| Number of High-Risk Samples (0.2 - 0.5) | 11 | 2 | 13 |
| Number of Very High/Extreme Risk Samples (<0.2) | 0 | 0 | 0 |

Note: MESS statistics reported here are based exclusively on the 201 spatially filtered occurrence points used for model calibration and validation. These values reflect the degree of environmental similarity between the occurrence localities and the training data. They do not characterize the full distribution of MESS values across all projected grid cells in the study area. Areas in the projected landscape with low MESS scores (i.e., environmentally novel relative to the training set) should be interpreted with caution.

Table S7. Net suitable habitat area changes under different thresholding approaches (Relative to 1970–2000 baseline)

| Threshold | 2021–2040 Net Loss (%) | 2041–2060 Net Loss (%) | Proportion of Loss in Marginal Zones (%) |
| --- | --- | --- | --- |
| Original classification (0.3 as lower bound) | 54 | 50 | 82 |
| Classification with ±0.05 shift (0.25 as lower bound) | 54 | 50 | 81 |
| Classification with ±0.05 shift (0.35 as lower bound) | 54 | 50 | 83 |
| MaxSSS threshold | 54 | 50 | 79 |
| 10% OMT threshold | 54 | 50 | 84 |

Note: Net loss expressed as percentage of the 1970–2000 baseline suitable habitat extent (396,000 km²). Marginal zones = low/moderate suitability areas (suitability score 0.3–0.7). The 54% near-term loss corresponds to approximately 214,000 km², and the 50% mid-century loss corresponds to approximately 198,000 km².

Table S8. Spatial distribution consistency of habitat contraction/expansion under different thresholding approaches

| Threshold | Primary Contraction Zones | Primary Expansion Zones | Spatial Overlap with Original Classification (%) |
| --- | --- | --- | --- |
| Original classification (0.3 as lower bound) | Qilian Mountains, Hengduan Mountains, Himalayan foothills | Tanggula Mountains, Kunlun Mountains | 100 |
| Classification with ±0.05 shift (0.25 as lower bound) | Qilian Mountains, Hengduan Mountains, Himalayan foothills | Tanggula Mountains | 90 |
| Classification with ±0.05 shift (0.35 as lower bound) | Qilian Mountains, Hengduan Mountains, Himalayan foothills | Tanggula Mountains, Kunlun Mountains | 93 |
| MaxSSS threshold | Qilian Mountains, Hengduan Mountains, Himalayan foothills | Tanggula Mountains | 92 |
| 10% OMT threshold | Qilian Mountains, Hengduan Mountains, Himalayan foothills | Tanggula Mountains, Kunlun Mountains | 94 |

Note: Spatial overlap = proportion of consistent contraction/expansion areas with original 0.3 threshold classification.


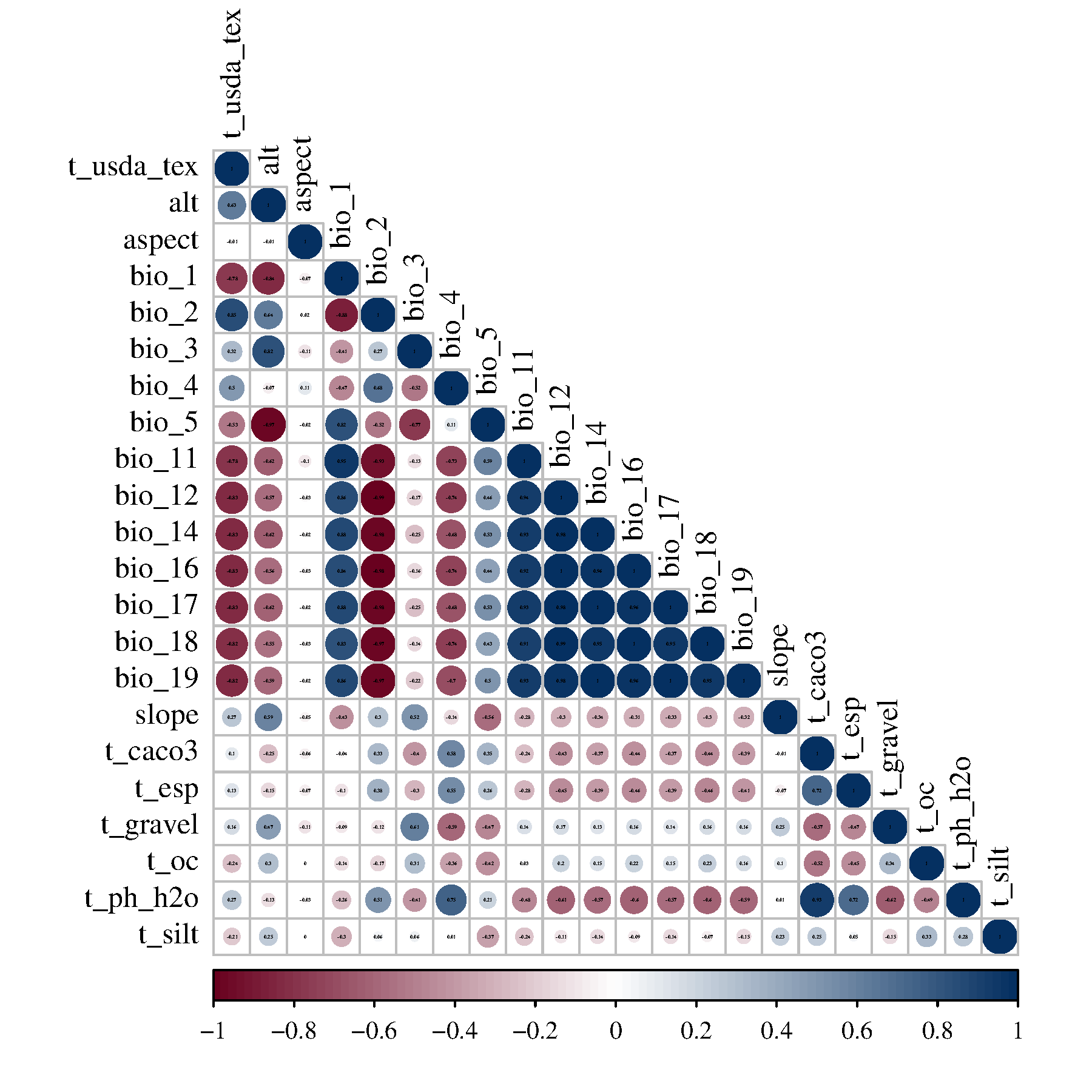


Figure S1 Spatial autocorrelation heat maps
